# Mitochondrial Dysfunction and Alterations in Mitochondrial Permeability Transition Pore (mPTP) Contribute to Apoptosis Resistance in Idiopathic Pulmonary Fibrosis Fibroblasts

**DOI:** 10.3390/ijms22157870

**Published:** 2021-07-23

**Authors:** Erika Rubí Luis-García, Carina Becerril, Alfonso Salgado-Aguayo, Omar Emiliano Aparicio-Trejo, Yair Romero, Edgar Flores-Soto, Criselda Mendoza-Milla, Martha Montaño, Victoria Chagoya, José Pedraza-Chaverri, Mohammed El Hafidi, Marisol Orozco-Ibarra, Annie Pardo, Moisés Selman

**Affiliations:** 1 Laboratorio de Biología Celular, Departamento de Fibrosis Pulmonar, Instituto Nacional de Enfermedades Respiratorias “Ismael Cosío Villegas”, Ciudad de México 14080, Mexico; erikarubi.84@gmail.com (E.R.L.-G.); lcbb6@hotmail.com (C.B.); criselda.mendoza@gmail.com (C.M.-M.); mamora572002@yahoo.com.mx (M.M.); 2 Laboratorio de Investigación en Enfermedades Reumáticas, Instituto Nacional de Enfermedades Respiratorias “Ismael Cosío Villegas”, Ciudad de México 14080, Mexico; alfonso.salgado@iner.gob.mx; 3 Departamento de Biología, Facultad de Química, Universidad Nacional Autónoma de México, Ciudad de México 04510, Mexico; emilianoaparicio91@gmail.com (O.E.A.-T.); pedraza@unam.mx (J.P.-C.); 4 Facultad de Ciencias, Universidad Nacional Autónoma de México, Ciudad de México 04510, Mexico; yair12@hotmail.com (Y.R.); apardos@unam.mx (A.P.); 5 Departamento de Farmacología, Facultad de Medicina, Universidad Nacional Autónoma de México, Ciudad de México 04510, Mexico; edgarfloressoto@yahoo.com.mx; 6 Departamento de Biología Celular y Desarrollo, Instituto de Fisiología Celular, Universidad Nacional Autónoma de México, Ciudad de México 04510, Mexico; vchagoya@ifc.unam.mx; 7 Departamento de Biomedicina Cardiovascular, Instituto Nacional de Cardiología, Ignacio Chávez, Juan Badiano 1, Belisario Domínguez Secc 16, Tlalpan, Ciudad de México 14080, Mexico; medelhafidi@yahoo.com; 8 Laboratorio de Neurobiología Molecular y Celular, Instituto Nacional de Neurología y Neurocirugía, Manuel Velasco Suárez, Av. Insurgentes Sur 3877, Colonia La Fama, Alcaldía Tlalpan, Ciudad de México 14269, Mexico; marisol.orozco.ibarra@gmail.com

**Keywords:** electron transport chain, IPF, mitochondrial dysfunction, mitochondrial networks, myofibroblasts

## Abstract

Idiopathic pulmonary fibrosis (IPF) is a devastating disease characterized by increased activation of fibroblasts/myofibroblasts. Previous reports have shown that IPF fibroblasts are resistant to apoptosis, but the mechanisms remain unclear. Since inhibition of the mitochondrial permeability transition pore (mPTP) has been implicated in the resistance to apoptosis, in this study, we analyzed the role of mitochondrial function and the mPTP on the apoptosis resistance of IPF fibroblasts under basal conditions and after mitomycin C-induced apoptosis. We measured the release of cytochrome c, mPTP opening, mitochondrial calcium release, oxygen consumption, mitochondrial membrane potential, ADP/ATP ratio, ATP concentration, and mitochondrial morphology. We found that IPF fibroblasts were resistant to mitomycin C-induced apoptosis and that calcium, a well-established activator of mPTP, is decreased as well as the release of pro-apoptotic proteins such as cytochrome c. Likewise, IPF fibroblasts showed decreased mitochondrial function, while mPTP was less sensitive to ionomycin-induced opening. Although IPF fibroblasts did not present changes in the mitochondrial membrane potential, we found a fragmented mitochondrial network with scarce, thinned, and disordered mitochondria with reduced ATP levels. Our findings demonstrate that IPF fibroblasts are resistant to mitomycin C-induced apoptosis and that altered mPTP opening contributes to this resistance. In addition, IPF fibroblasts show mitochondrial dysfunction evidenced by a decrease in respiratory parameters.

## 1. Introduction

Idiopathic pulmonary fibrosis (IPF) is an aging-associated, progressive, and irreversible disease of unknown etiology. Currently, therapeutic options remain limited, and there is no efficient treatment to improve expectancy and quality of life [1,2]. Furthermore, the pathogenic mechanisms have not been fully elucidated. However, strong evidence suggests that IPF begins with micro-lesions and activation of the alveolar epithelium, which secretes various mediators that induce migration, proliferation, and activation of fibroblasts that produce excessive extracellular matrix amounts with progressive destruction of the lung parenchyma [3,4]. An intriguing aspect of the pathogenesis of IPF is the resistance shown by fibroblasts and myofibroblasts to apoptosis, while on the contrary, alveolar epithelial cells are very susceptible to this process [3,4,5]. The mechanisms of this paradoxical behavior are uncertain, but in the case of epithelial cells, their susceptibility has been attributed, at least partially, to mitochondrial dysfunction [5].

Fibroblasts and myofibroblasts are the critical effector cells in tissue fibrosis due to their participation in elaborating the extracellular matrix and contraction mechanisms and remodeling of damaged tissue [6]. During physiological regeneration, fibroblasts are eliminated by apoptosis, and evasion or resistance to this process has been associated with progressive fibrosis. In this context, it has been reported that fibroblasts and myofibroblasts from patients with IPF are resistant to apoptosis, although the molecular mechanisms involved are uncertain [7,8,9,10]. Due to the central role that mitochondria play in the implementation, amplification, and regulation of cellular apoptosis, this organelle would be involved in the apoptosis resistance of IPF fibroblasts, but this has not yet been studied in depth.

Mitochondria are central organelles that participate in vital metabolic processes, synthesize most ATP, and regulate several signaling cascades, including apoptosis [11]. The transduction of apoptotic signaling of cell death requires permeabilization of the mitochondrial membrane and the subsequent release of pro-apoptotic factors from the mitochondrial inter-membrane space (IMS) (i.e., cytochrome c, apoptosis-binding protein with low pI (Smac/Diablo), Endonuclease G, Serine protease Htra2 (Htra2/Omi), and apoptosis-inducing factor (AIF)) [12]. The release of cytochrome c from mitochondria is commonly considered as the “point-of-no-return” in the sequence of events leading to apoptosis and involves changes in the mitochondrial membrane permeability transition (mtMPT) [13]. mtMPT results in the drop of the mitochondrial membrane potential (Δψm), osmotic swelling of the mitochondrial matrix, rupture of the outer mitochondrial membrane, and release of cytochrome c [14].

The mitochondrial permeability transition pore (mPTP) is a non-specific voltage-dependent channel that forms in the inner mitochondrial membrane. The molecular nature of mPTP is controversial due to the lack of knowledge about the identity of the proteins that form it. There are different hypotheses about the components that constitute the mPTP. One of them suggests the participation of the proteins adenine nucleotide translocator (ANT), voltage-dependent anion-selective channel (VDAC), and cyclophilin D (CypD), suggesting that ANT/VDAC form the basic unit of the mPTP, and that the recruitment of additional proteins, such as CypD, Bax, hexokinase, or translocator protein (TSPO), modulate the activation of the mPTP. Another hypothesis recently developed has implicated different subunits of the F_1_F_0_–ATP synthase as the inner membrane pore-forming unit of the mPTP [15,16].

Urbani et al. [17] proposed that active F_1_F_0_-ATP synthase is responsible for the formation of mPTP; they used highly purified F_1_F_0_-ATP synthase and showed that calcium treatment mimics the response with specific agonists and inhibitors of mPTP. Recently, Pinke and Sazanov [18] also stated that the entire mammalian ATP synthase is part of the mPTP, based on an atomic model of ATP opening developed through cryogenic electron microscopy (cryo-EM) data. Despite these findings, the formation of the mPTP from F_1_F_0_-ATP synthase has been questioned in recent studies, where some authors showed that mPTP persists in the absence of several subunits of F_1_F_0_-ATP synthase. Since deleting such subunits prevents the assembly of functional F_1_F_0_-ATP synthase, the authors concluded that ATP synthase does not participate directly in the mPTP formation [19,20].

mPTP opening is triggered by matrix Ca^2+^, but its activity can be modulated by several other factors such as oxidative stress, adenine nucleotide depletion, high inorganic phosphate (Pi) concentrations, mitochondrial membrane depolarization, or uncoupling [21]. The prolonged mPTP opening leads to an abrupt increase in the permeability of the inner mitochondrial membrane to solutes with molecular mass up to 1.5 kDa, provoking mitochondrial depolarization, followed by respiratory inhibition and the generation of reactive oxygen species (ROS), and massive release of matrix Ca^2+^.

This event induces the swelling of mitochondria, leading to breaks in the outer mitochondrial membrane that induce the release of pro-apoptotic factors [22]. For this reason, the mPTP has been proposed as a pivotal effector in the process of cell death [23,24].

Based on this accumulating evidence, we hypothesized that the resistance of IPF fibroblasts to apoptosis might be associated with a dysregulation in the mitochondrial function, mainly by resistance to the mPTP opening. Therefore, we aimed to analyze the mitochondrial function and the mPTP opening on the apoptosis resistance of human IPF fibroblasts. In the present study, we demonstrate that in IPF fibroblasts, mitochondria are dysmorphic and dysfunctional and that the resistance to apoptosis is related to altered mPTP opening. These findings may provide novel insights into the mechanisms involved in the resistance to apoptosis by IPF fibroblasts.

## 2. Results

### 2.1. IPF Fibroblasts Are Resistant to Mitomycin C-Induced Apoptosis

**Figure 1:**
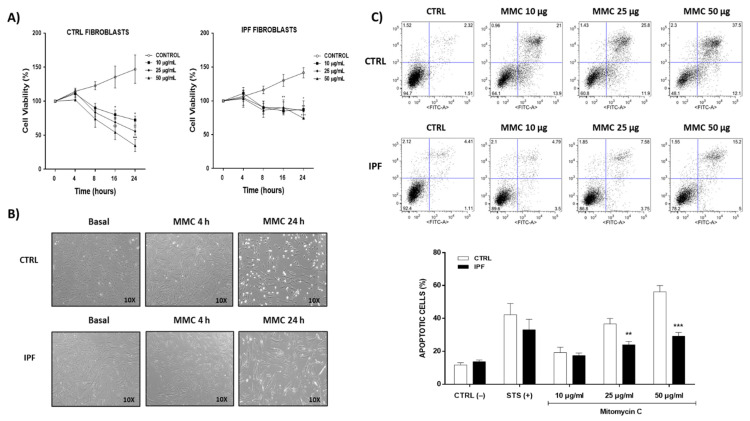
Effect of mitomycin C (MMC) on viability and resistance to apoptosis in idiopathic pulmonary fibrosis and normal lung fibroblasts. (**A**) After treatment with increasing concentration of mitomycin C for 4, 8, 16, and 24 h, the effect on cell viability was determined using the WST-1 assay. The data represent the means ± SEM of two independent experiments ** *p* < 0.01, *** *p* < 0.01, compared with the control group. (**B**) Bright field representative images from IPF and control fibroblasts after incubation with mitomycin C (25 μg/mL) for 4 and 24 h. (**C**) Cell apoptosis rates were analyzed via flow cytometry using the Annexin V/propidium iodide (FITC/PI) double staining method. Fibroblasts were stimulated with mitomycin C for 24 h. Staurosporine (STS, 1 μM) was used as a positive control. The results are expressed as the mean ± SEM. The differences were analyzed using two-way ANOVA followed by the Bonferroni multiple comparisons test. ** *p* < 0.05 or *** *p* < 0.01 vs. control. CTRL (*n* = 2) and IPF (*n* = 4).

Mitomycin C has been shown to inhibit fibroblast proliferation and to induce apoptosis through activation of the intrinsic mitochondrial pathway in human fibroblasts when administered in high doses [25,26]. To investigate the effect of mitomycin C on fibroblasts, we evaluated cell viability using the WST-1 assay following exposure to various mitomycin C concentrations (10, 25, or 50 μg/mL) for different times (4, 8, 16, or 24 h). As shown in Figure 1A, mitomycin C significantly reduced the viability of control fibroblasts in a dose- and time-dependent manner. Treatment of control fibroblasts with mitomycin C 25 μg/mL for 24 h led to a nearly 50% decrease in cell viability. In contrast, mitomycin C did not produce the same effect in IPF fibroblasts. The maximum percentage of cell death was reached with mitomycin C 10 μg/mL at 8 h, and no further cell death was reached by increasing the incubation time or mitomycin C concentration. Representative bright-field micrographs using 25 μg/mL mitomycin C are shown in Figure 1B. To quantitatively examine the effect of mitomycin C on apoptosis, Annexin V-FITC/PI double staining was measured via flow cytometry in IPF and control fibroblasts. Following treatment with mitomycin C (10, 25, and 50 μg/mL at 24 h), the percentage of apoptotic cells (including early and late apoptosis) was evaluated. The results showed that IPF fibroblasts are significantly resistant to apoptosis induced by mitomycin C at 25 and 50 μg/mL, compared with control fibroblasts (** *p* ˂ 0.05 or *** *p* ˂ 0.01) Figure 1C.

### 2.2. IPF Fibroblasts Show a Decrease in the Mitomycin C-Induced Release of Cytochrome C

**Figure 2:**
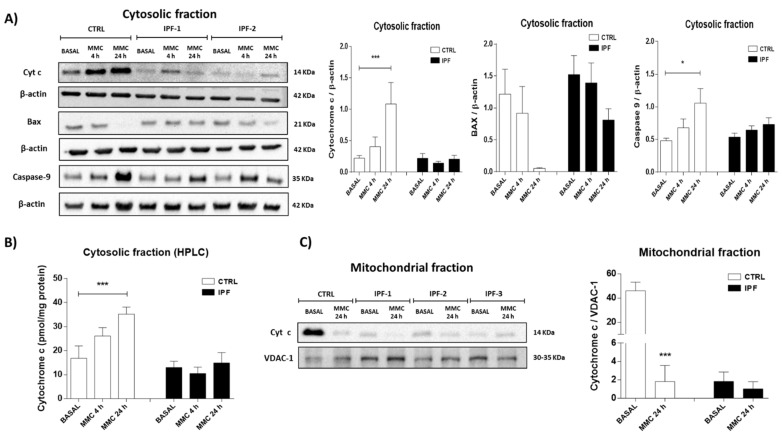
Fibroblasts from idiopathic pulmonary fibrosis show a decrease in the release of cytochrome c induced for mitomycin C (MMC). After exposure to 25 μg/mL mitomycin C for 4 and 24 h, we isolated the cytosolic and mitochondrial fractions in control and IPF fibroblasts. (**A**) Expression levels of cytochrome c, caspase 9, and Bax in cytosolic fraction were determined via Western blot analysis. The expression levels of β-actin were used as a loading control. (**B**) Cytosolic fraction was also analyzed by the high-performance liquid chromatography (HPLC) technique. (**C**) The expression levels of cytochrome c in mitochondrial fraction were determined via Western blot analysis. Expression levels of VDAC-1 were used as a loading control. The results are expressed as the mean ± SEM of at least three independent experiments. The differences were analyzed using one-way ANOVA followed by Dunnett’s multiple comparisons test, * *p* < 0.05, *** *p* < 0.01 vs. control, CTRL (*n* = 3) and IPF (*n* = 3).

To determine whether the resistance to apoptosis in IPF fibroblasts is associated with the release of cytochrome c, IPF and control fibroblasts were stimulated with mitomycin C (25 μg/mL) for 4 and 24 h. Subsequently, the cytosolic and mitochondrial fractions were isolated. The release of cytochrome c was determined in the cytosolic fraction by Western blot (WB) and HPLC, while the mitochondrial fraction was only analyzed by WB due to the small amount of sample obtained during isolation. As illustrated in Figure 2A, the cytosolic fraction isolated from control fibroblasts had a higher cytochrome c content after mitomycin C treatment than basal condition (*** *p* < 0.01). In contrast, the cytosolic cytochrome c content did not change in IPF fibroblasts after mitomycin C treatment. These results were confirmed by HPLC analysis (Figure 2B). Additionally, we analyzed the expression levels of Bax, a Bcl-2-associated X protein. We found a strong decrease of Bax in the cytosolic fraction of control fibroblasts stimulated with mitomycin C for 24 h, suggesting that Bax was translocated from the cytosol to the mitochondrial membrane, contributing to the apoptosis process. By contrast, the level of this protein showed only a slight decrease at 24 h in IPF fibroblasts. Moreover, the stimulation with mitomycin C for 24 h increased the active form of caspase 9 in the cytosolic fraction of control fibroblasts (* *p* ˂ 0.05) but not in IPF fibroblasts (Figure 2A). These results show that the intrinsic pathway is activated in control fibroblasts but not in IPF fibroblasts.

On the other hand, the analysis of the mitochondrial fraction of control fibroblasts showed a high content of cytochrome c under basal conditions, while the mitomycin C treatment for 24 h provoked a significant decrease (*** *p* ˂ 0.01). Interestingly, under basal conditions, the mitochondrial cytochrome c content in IPF fibroblasts was significantly lower than in control fibroblasts, while treatment did not change their levels (Figure 2C). As a whole, these data indicate that IPF fibroblasts released fewer cytochrome c after stimulation with 25 μg/mL mitomycin C at 24 h compared with control fibroblasts.

### 2.3. IPF Fibroblasts Show Strong Resistance to mPTP Opening Induced By Ionomycin

**Figure 3:**
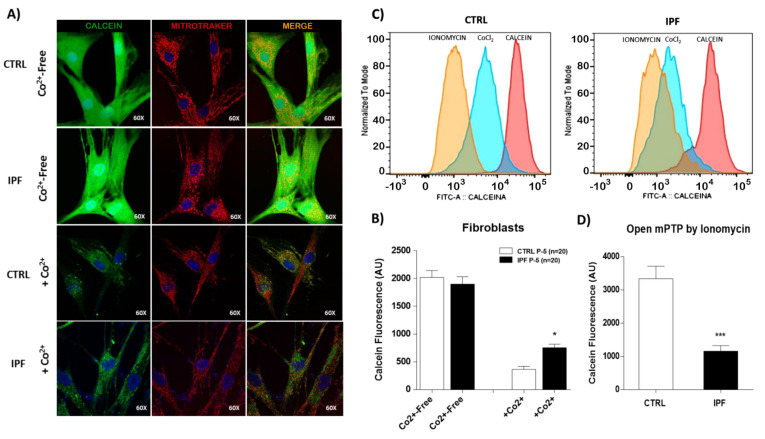
Idiopathic pulmonary fibrosis fibroblasts show strong resistance to ionomycin-induced mPTP opening. (**A**) Representative confocal micrographs of IPF and control fibroblasts after incubation with calcein-AM (1 μM) and MitoTracker Red (150 nM) in the presence or absence of Co^2+^ (1 mM), which quenches calcein fluorescence (green) outside of mitochond ria. In the absence of Co^2+^, the calcein fluorescent signal is very intense and is present in the entire cell. The calcein fluorescence in mitochondria is lower in the presence of Co^2+^. (**B**) The bar graph shows the quantification of calcein fluorescence in IPF and control cells in the absence or presence of Co^2+^. The number shown in the panel indicates the number of cells used in the study. The results are expressed as the mean ± SEM. The differences were analyzed using one-way ANOVA followed by the Bonferroni multiple comparisons test. * *p* < 0.05. (**C**) Representative flow cytometry histogram shows the intensity of calcein signals in IPF and control fibroblasts. The change in fluorescence between Co^2+^ and ionomycin indicates the continuous activation of mitochondrial permeability transition pores. (**D**) The bar graph shows the quantification of mean calcein fluorescence in IPF and control cells. The results are expressed as the mean ± SEM. The differences were analyzed using an unpaired *t*-test. *** *p* < 0.01 vs. control. CTRL (*n* = 3) and IPF (*n* = 4).

**Figure 4:**
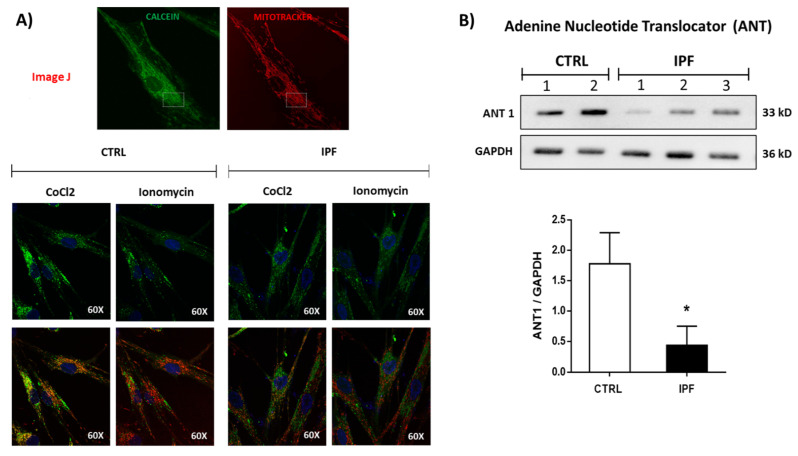
Idiopathic pulmonary fibrosis fibroblasts show resistance to mPTP opening induced by ionomycin. (**A**) Representative confocal micrographs of IPF and control fibroblasts after incubation with calcein-AM (1 μM), MitoTracker Red (150 nM), and Co^2+^(1 mM), in the presence or absence of ionomycin (1 μM), which quenches calcein fluorescence (green) within mitochondria. The fluorescence of calcein is maintained in IPF fibroblasts after ionomycin administration compared to control fibroblasts. (**B**) Image representative of ANT protein expression in control and IPF fibroblasts. The expression levels of GAPDH were used as a loading control. The results are expressed as the mean ± SEM. The differences were analyzed using an unpaired *t*-test. * *p* < 0.05 vs. control, CTRL (*n* = 2) and IPF (*n* = 3), one experiment.

It has been proposed that modulation of mPTP opening is a strategic regulator of cell death by apoptosis. We determined whether mPTP opening was associated with the apoptosis resistance shown by IPF fibroblasts. We compared the opening of mPTP in control and IPF fibroblasts under basal conditions using the CoCl_2_-calcein fluorescence-quenching assay. First, we measured the calcein fluorescence by confocal microscopy in the absence of cobalt. As expected, calcein fluorescence is much higher in the absence of CoCl_2_ (Co^2+^-free). The results showed similar levels of calcein between IPF and normal lung fibroblasts, indicating similar calcein loading (Figure 3A,B). However, in the presence of CoCl_2_, calcein fluorescence inside mitochondria is lower in control than in IPF fibroblasts (* *p* ˂ 0.05). This result suggests that mPTP is more open in controls than IPF fibroblasts (Figure 3A,B). We also analyzed mPTP opening in fibroblasts by flow cytometry using a MitoProbe Transition Pore Assay Kit to corroborate this finding. The change in fluorescence between cells incubated with only CoCl_2_ and those incubated with ionomycin indicates the continuous activation of mPTP. Our results showed that mPTP opening induced by ionomycin was higher in control than in IPF fibroblasts (*** *p* ˂ 0.01 vs. control) (Figure 3C,D). This result confirmed that IPF fibroblasts are most resistant to mPTP opening ionomycin-induced, as demonstrated by the retention of calcein fluorescence in the presence of ionomycin in IPF fibroblasts (Figure 4A). We also examined the expression levels of ANT-1 protein, and as shown in Figure 4B, these were markedly lower in IPF compared with controls fibroblasts (* *p* < 0.05 vs. control). This result is captivating because this protein participates in mPTP formation.

### 2.4. IPF Fibroblasts Show Decreased Release Mitochondrial Calcium 

**Figure 5:**
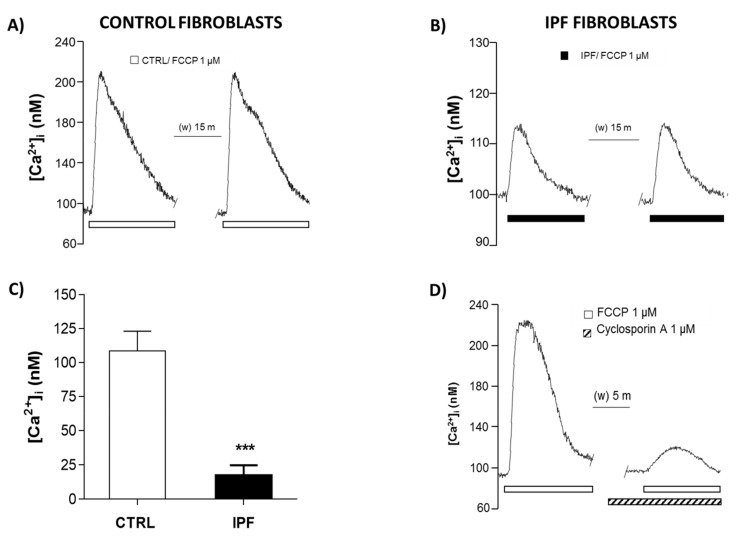
Typical recordings of simultaneous intracellular calcium concentration assay ([Ca^2+^]i) control fibroblasts and IPF. Two pulses of FCPP were applied with an interstimulus interval of 15 min to determine the response reproducibility (**A**,**B** respectively) (CTRL *n* = 2; IPF *n* = 2). Six fibroblasts were evaluated for each experimental condition. (**C**) Data are shown as media ± SEM and were compared by Student’s *t*-test, *** *p* < 0.01. (**D**) Ca^2+^ increase induced by FCCP was blocked by cyclosporin A (*n* = 2).

We also evaluated mitochondrial calcium in control and IPF fibroblasts, since elevated mitochondrial calcium levels induces the opening of the mPTP. We used FCCP to increases the [Ca^2+^]_i_ by activating the mPTP in control and IPF fibroblasts. Our results indicate that FCCP elicited in normal fibroblasts a transient moderate increase in [Ca^2+^]_i_ to 115.76 ± 9.53 and 108.09 ± 7.01 nM, respectively (Figure 5A). In contrast, the IPF fibroblasts exhibited a significantly diminished increase in [Ca^2+^]_i_ levels by 13.59 ± 1.49 and 18.36 ± 5.24 nM, respectively (Figure 5B,C) (*** *p* ˂ 0.01). To corroborate that the increase in [Ca^2+^]_i_ is due to the activation of the mPTP with FCCP, we blocked pharmacologically the mPTP with cyclosporin A, inhibiting the response (Figure 5D).

### 2.5. Inhibition of the mPTP Opening with BKA Decreased Apoptosis in Control Fibroblasts

**Figure 6:**
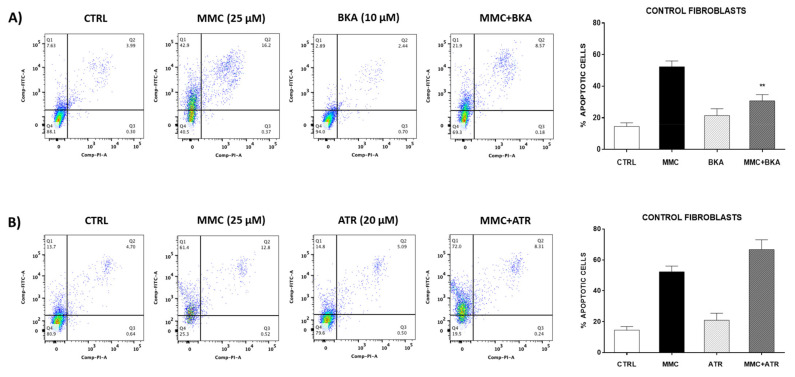
Control fibroblasts show resistance to apoptosis induced by mitomycin C in the presence of bongkrekic acid (BKA) without changes with atractyloside (ATR). (**A**) Representative image of dot plot analysis by flow cytometry. Cell apoptosis rates were analyzed using the Annexin V-FITC/PI double-staining method. (**B**) Fibroblasts were stimulated with BKA (10 μM) for 6 h, and then, we incubated fibroblasts with mitomycin C MMC (25 μg/mL) for 24 h. Fibroblasts were stimulated with ATR (20 μM) for 1 h, and then, we incubated fibroblasts with (25 μg/mL) for 24 h. The results are expressed as the mean ± SEM. The differences were analyzed using one-way ANOVA followed by the Bonferroni multiple comparisons test, ** *p* < 0.01 vs. control. CTRL (*n* = 3).

To determine whether the inhibition of mPTP opening participates in the resistance of fibroblasts to apoptosis, control fibroblasts were stimulated with BKA (10 μM, for 6 h), an inhibitor of the opening of mPTP, and then were stimulated with mitomycin C (25 μg/mL) for 24 h. Our results showed an increase in the number of positive apoptotic cells when we used mitomycin C. Interestingly, the administration of BKA decreased the percentage of apoptotic cells in control fibroblasts (** *p* ˂ 0.01), which are very susceptible to cell death (Figure 6A). On the other hand, when we stimulated control fibroblasts with ATR, an inducer of mPTP opening, we found a slight and not significant increase in the percentage of apoptosis induced by mitomycin C (Figure 6B).

### 2.6. IPF Fibroblasts Exhibit Lower Oxygen Consumption Than Control Fibroblasts

**Figure 7:**
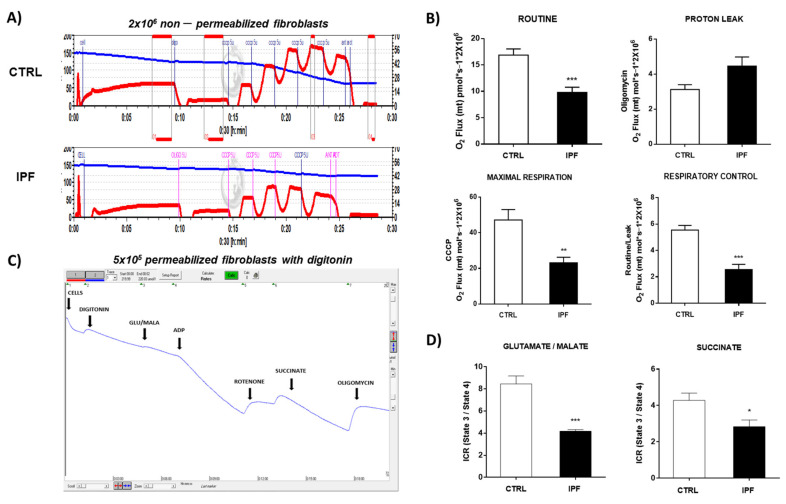
Oxygen consumption in idiopathic pulmonary fibrosis and control fibroblasts with and without induction of permeability. Intact fibroblasts were placed in a 2 mL chamber at a final concentration of 2 × 10^6^ cells/mL. Measurement of oxygen consumption was performed at 37 °C in high-resolution oxygraphy (Oxygraph-2k Oroboros Instruments, Innsbruck, Austria). Proton leak was induced by oligomycin (5 μM), and titrations of 1 μM CCCP induced the maximum uncoupled respiration. Finally, we inhibit the OXPHOS with rotenone and antimycin. (**A**) Representative traces of oxygen consumption in control and IPF fibroblasts. (**B**) Graphs of different parameters of mitochondrial respiration (Routine, Proton Leak, Maximal Respiration, and Respiratory Control). The results are expressed as the mean ± SEM. The differences were analyzed using an unpaired *t*-test, ** *p* < 0.05, *** *p* < 0.01 vs. control. CTRL (*n* = 2) and IPF (*n* = 4). (**C**) Representative traces of oxygen consumption using different substrates for complex I and II. (**D**) Graphs of respiratory control index (ICR) using glutamate/malate (10 mM/5 mM) and succinate (5 mM). Respiration inhibition was carried out in the presence of rotenone (0.5 μM) by complex I and oligomycin (5 μM) complex II. The results are expressed as the mean ± SEM. The differences were analyzed using an unpaired *t*-test, * *p* < 0.05, *** *p* < 0.01 vs. control. CTRL (*n* = 3) and IPF (*n* = 2).

To investigate whether apoptosis resistance in IPF fibroblasts is associated with mitochondrial dysfunction, we analyzed the integrity of mitochondria through oxygen consumption in IPF and control fibroblasts. Oxygen flux (pmol O_2_/s/10^6^ cells/mL) was determined in 2 × 10^6^ non-permeabilized fibroblasts. Figure 7A shows a representative trace of control and IPF fibroblasts respiration. The routine respiration of fibroblasts was measured in standard incubation media (10–20 min), and then, we performed sequential injections of substrate–uncoupler–inhibitor–titration for key parameters of mitochondrial respiration. As shown in Figure 7B, routine respiration of control fibroblasts was higher than IPF fibroblasts (*** *p* ˂ 0.01), which was suppressed by oligomycin, a specific inhibitor of mitochondrial ATP synthase and oxidative phosphorylation. The proton leak was slightly increased in the IPF fibroblasts, but the difference against control fibroblasts was not significant (*p* = 0.06). The maximal respiration of fibroblasts was determined after the uncoupling of mitochondria with CCCP. The results showed that IPF fibroblasts had a decreased maximal respiration compared with the control group (** *p* ˂ 0.01). Finally, respiratory control (RC) was calculated by the difference between basal respiration and proton leak. The results showed that IPF fibroblasts exhibited lower RC than normal fibroblasts (*** *p* ˂ 0.01). Together, our findings indicate impaired respiration in IPF fibroblasts. We evaluated the oxygen consumption in permeabilized fibroblasts to determine whether the modification of respiration in IPF fibroblasts was associated with an alteration in complex I and II of the electron transport chain. We determined the electron transport chain activity using specific substrates to complex I (glutamate/malate) and complex II (succinate). A representative trace is shown in Figure 7C. The electron transport chain activity was decreased in IPF fibroblasts when it was stimulated through complex I (** *p* ˂ 0.01) or complex II (* *p* ˂ 0.05) (Figure 7D).

### 2.7. The Mitochondrial Transmembrane Potential Is Similar in IPF and Control Fibroblasts 

**Figure 8:**
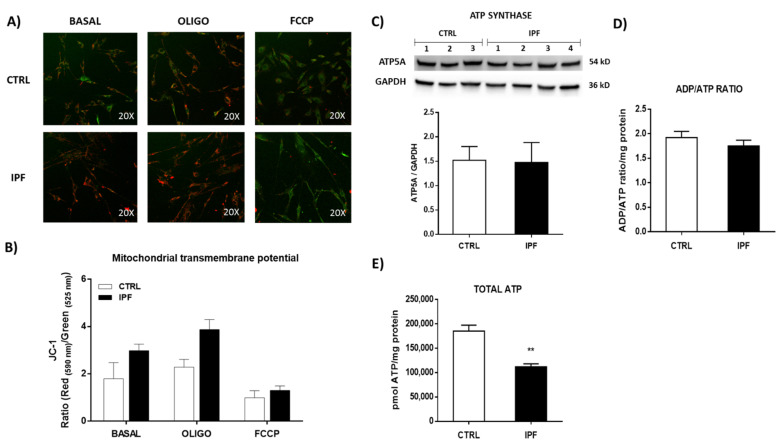
IPF fibroblasts do not modify the mitochondrial transmembrane potential or the ADP/ATP ratio but decrease the total levels of ATP. (**A**) Representative fluorescence microscopic image of control and IPF fibroblasts after staining with JC-1 (4 μm) in the presence or absence of oligomycin (1 μM) or FCCP (10 μM). The intensity of JC-1 reflects the level of mitochondrial transmembrane potential. (**B**) The bar graph shows the quantification of JC-1 signals in control and IPF fibroblasts in the presence or absence of oligomycin and FCCP using fluorescence microscopy. The results are expressed as the mean ± SEM. CTRL (*n* = 1) and IPF (*n* = 2) (**C**) Expression levels of ATP synthase in the lysate of control and IPF fibroblasts were determined via Western blot analysis. The expression levels of GAPDH were used as a loading control CTRL (*n* = 3) and IPF (*n* = 4) from three independent experiments. (**D**) ADP/ATP ratio was quantified by a luciferin/luciferase-based luminescence assay. The results are expressed as the mean ± SEM. The differences were analyzed using an unpaired *t*-test. CTRL (*n* = 4) and IPF (*n* = 4). (**E**) ATP production was quantified by luminescence assay in control and IPF fibroblasts. The results are expressed as the mean ± SEM. The differences were analyzed using an unpaired *t*-test. ** *p* < 0.01 vs. control. CTRL (*n* = 3) and IPF (*n* = 3).

As we detected alterations in oxygen consumption, we turned our attention toward the mitochondrial membrane potential: the electrochemical force that modulates the kinetics of proton re-entry to the matrix through ATP synthase. Using confocal microscopy, we measured the ΔΨm of control and IPF fibroblast stained with JC-1 (4 μM). JC-1 is a cationic fluorescent dye that exhibits potential-dependent accumulation in mitochondria (Figure 8A). We did not find significant differences in the JC-1 fluorescence signal between control and IPF fibroblasts (Figure 8B). To ensure that dye is equally loaded and that the JC-1 signal is not auto-quenched, we compared JC-1 fluorescence in control and IPF fibroblasts following oligomycin or FCCP treatment. Oligomycin, an inhibitor of ATP synthase, induces hyperpolarization of mitochondria and increases JC-1 fluorescence, whereas FCCP dissipates transmembrane potential (Figure 8B).

### 2.8. IPF Fibroblasts Display Decreased ATP Levels but Do Not Show a Change in ADP/ATP Ratio

Diverse studies have proposed the participation of ATP synthase in the formation of mPTP. For this reason, we evaluated the levels of expression of ATP synthase by Western blot in control and IPF fibroblasts, and no differences were found (Figure 8C). Furthermore, we evaluated the ADP/ATP ratio in control and IPF fibroblasts, and also, no significant differences were observed, suggesting no changes in ATP synthase activity (Figure 8D).

ATP production in IPF and control fibroblasts was determined quantitatively through a luminescence assay. The results showed that IPF fibroblasts had a lower ATP total concentration than the control group (** *p* ˂ 0.01). In addition, decreased mitochondrial respiration in IPF fibroblasts was correlated with a decrease in ATP production (Figure 8E).

### 2.9. IPF Fibroblasts Display Abnormal Mitochondrial Structure

**Figure 9:**
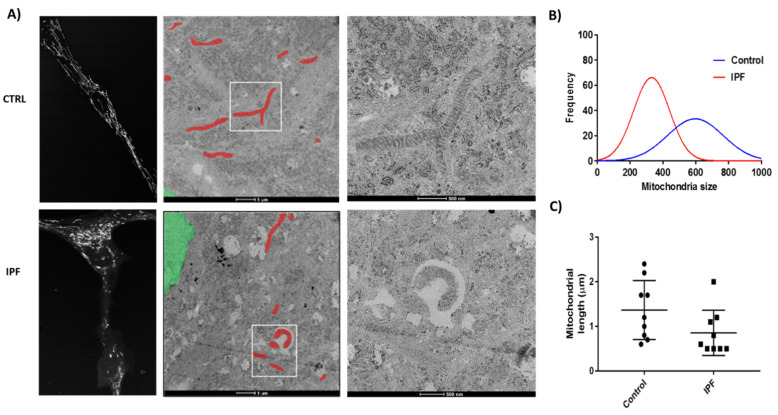
Number and morphology of lung fibroblasts mitochondria from healthy donors and idiopathic pulmonary fibrosis patients. (**A**) Representative confocal and transmission electron microscopy of mitochondrial morphology of lung fibroblasts derived from healthy donors and IPF patients. Two different magnifications of the same cell are shown, and mitochondria are highlighted in red. (**B**) Frequency histogram of mitochondria size (nm^2^) *n* > 100, using at least five different cells of each condition CTRL (*n* = 2) and IPF (*n* = 2). (**C**) Images were semi-automatic analyzed using the ImageJ software. The length of mitochondria was used to determine mitochondrial elongation mean per image; at least 70 mitochondria per condition were measured.

In order to evaluate the mitochondrial structure, we analyzed IPF and control fibroblasts by fluorescence and transmission electron microscopy (TEM). As shown in the fluorescence micrograph with MitoTracker, we observed a mitochondrial network fragmented and accumulation of mitochondria in IPF compared to control fibroblasts. Moreover, fibroblasts from control lungs showed branched, elongated, and lengthwise organized mitochondria with a typical appearance; the cristae were electrodense and compact. In contrast, in IPF fibroblasts, the mitochondria were scarce, thinned, and disordered; the cristae showed a lesser electron density than control fibroblasts (Figure 9A). As shown in the frequency histogram, control fibroblasts mitochondria have a size of around 600 nm^2^, while mitochondria from patients with IPF have a much smaller size of about 250 nm^2^ (Figure 9B). We also evaluated the mitochondrial elongation by the length of mitochondria mean per image using the ImageJ software. The IPF mitochondria exhibited lower elongation than mitochondrial control (Figure 9C).

**Figure 10:**
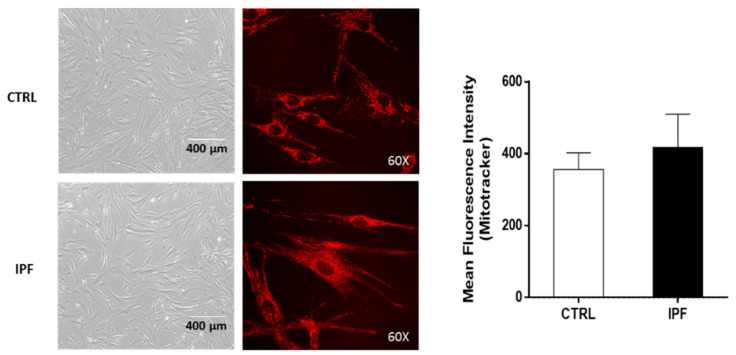
Idiopathic pulmonary fibrosis fibroblasts do not show differences in mean fluorescence intensity of MitoTracker compared to control fibroblasts (whole cells). To determine indirectly if IPF fibroblasts had a lower number of mitochondria than control fibroblasts, using a confocal microscope, we performed an analysis of the mean total fluorescence intensity of MitoTracker in a Z-stack of complete fibroblasts. The results are expressed as the mean ± SEM. The differences were analyzed using an unpaired *t*-test, * *p* < 0.05 vs. control. CTRL (*n* = 2) and IPF (*n* = 2).

We also determined by confocal microscopy, through a Z-stack analysis, the mean fluorescence intensity of the MitoTracker in whole cells in control and IPF fibroblasts. In addition, sections were made throughout the entire cell to indirectly assess whether there were changes in mitochondrial mass. Our results show no significant change in the fluorescence of the mitochondrial marker MitoTracker between control and IPF fibroblasts, suggesting that there are no significant differences in the number of mitochondria (Figure 10). In addition, previous studies have shown that there are no differences in the amount of mitochondrial DNA between normal lung and IPF fibroblasts [5].

## 3. Discussion

A growing body of evidence demonstrates that IPF fibroblasts are resistant to apoptosis [7,8,9,10]. The mechanisms are unclear, but evidence indicates the participation of the extrinsic pathway of apoptosis, such as the inhibition of Fas-mediated apoptotic cell death [8]. However, no studies have investigated the role of the critical intrinsic pathway of apoptosis mediated by mitochondria. In this context, our study demonstrates that IPF fibroblasts show resistance to mPTP opening, low cytochrome c levels, mitochondrial dysfunction, and a fragmented mitochondrial network. Recently, mitochondrial dysfunction has been reported in IPF fibroblasts associated with the induction and maintenance of the senescent phenotype [27], but its specific role in the apoptosis of these cells is uncertain. Mitochondria-induced apoptosis arises from various internal cell stresses, resulting in the release of cytochrome c toward the cytosol. The above is a crucial process in the intrinsic apoptosis pathway and is related to the mPTP opening. Once in the cytosol, cytochrome c activates the apoptosome formation, which is a cytosolic multiprotein complex composed of cytochrome c, Apaf-1, and ATP. This complex induces the caspase 9 activation, triggering the effector caspases activation that provokes cell death [28]. Therefore, in the fibrotic process, the dysregulation of cytochrome c release can result in the accumulation of apoptosis-resistant fibroblasts, excessive extracellular matrix deposition, and disruption of the lung architecture.

In this work, we demonstrated that IPF fibroblasts presented a marked decrease in basal cytochrome c levels and its release after stimulation with mitomycin C. Cytochrome c is a crucial signaling molecule during apoptosis, but it also plays an essential role in oxidative phosphorylation by transferring electrons from complex III to complex IV of the electron transport chain [29]. A substantial portion of cytochrome c is cardiolipin-associated, embedded in mitochondrial cristae, while the remaining cytochrome c is free in the intramembrane space. The release of cytochrome c from mitochondria is carried out during the early apoptosis stages and requires a two-step process. First, the solubilization of cytochrome c involves a breaking of the electrostatic and hydrophobic forces that it usually maintains with cardiolipin. Second, the mitochondrial permeability transition is sufficient to release cytochrome c into the cytosol [30]. Thus, the decrease in basal cytochrome c levels observed in fibroblasts from patients with IPF may be related to decreased electron transfer, oxygen consumption, and ATP synthesis. In this context, various studies have reported that the lack of cytochrome c disrupts the assembly and stability of respiratory complexes I and IV in fibroblasts [31].

Interestingly, embryonic fibroblasts derived from mice deficient in the somatic isoform of cytochrome c are resistant to apoptosis by agents known to trigger the intrinsic apoptotic pathway. This effect was associated with respiratory chain defects [32]. The mitochondrial apoptosis pathway is regulated mainly by changes in the permeability of the mitochondrial membrane or by alterations in its function. Mitochondrial outer membrane permeabilization is the ultimate step of apoptotic signal transduction pathways, which converge on mitochondria. mPTP is one of the representative systems proposed to be responsible for mitochondrial outer membrane permeabilization [33]. Although the concept of the mPTP is still evolving, mounting evidence indicates that the mPTP is directly responsible for cytochrome c release. For this reason, it is considered a strategic regulator of cell death. The mPTP is a non-specific conductance channel in the inner mitochondrial membrane that allows the flux of metabolites with a molecular weight of up to 1.5 kDa.

Induction of the mPTP leads to mitochondrial depolarization, inhibition of ATP synthesis, Ca^2+^ uptake, respiratory inhibition, generation of ROS, mitochondrial swelling, and potentially the rupture of the outer mitochondrial membrane leading to release of mitochondrial apoptogenic proteins such as cytochrome c, Smac/DIABLO, endonuclease G, and AIF. Therefore, it is not surprising that studies of the mPTP attract substantial attention. However, despite significant effort, the exact molecular composition of the mPTP is still a matter of debate.

The mPTP opening can be induced by high concentrations of mitochondrial Ca^2+^, oxidative stress, and Pi and can be inhibited by cyclosporin A, adenine nucleotides, Mg^2+^, acidic pH, and reducing agents in the cells [16]. The mPTP has been implicated in several diseases, but to our knowledge, no studies had been performed on IPF fibroblasts. We demonstrated for the first time that IPF fibroblasts are resistant to ionomycin-induced mPTP opening; this is an intriguing finding, because high basal ROS production by IPF fibroblasts has been reported [34], and an mPTP opening should be expected as a consequence of high mitochondrial ROS production. However, a similar effect has been reported in cancer cells. For example, Norman et al. demonstrated that mPTP inhibition by cyclosporin A promotes skin cancer in transplanted patients by allowing keratinocyte survival under conditions of genotoxic stress, highlighting the critical role of mPTP inhibition in tumor development [35].

On the other hand, different factors can influence this resistance to ionomicyn-induced mPTP opening, such as the expression of protein components. Recently, it has been proposed that in addition to the ANT, VDAC, cyclophilin D, the ATP synthase is part of the mPTP [17,18].

However, this idea has been questioned in recent studies, where some authors showed that mPTP persists in the absence of several subunits of ATP synthase [19,20]. We analyzed the expression levels of ATP synthase by WB and did not find significant differences between control and IPF fibroblasts, suggesting that the content of ATP synthase cannot influence the function of mPTP. Interestingly, when we analyzed the expression of ANT, we found that this protein shows decreased levels in IPF fibroblasts, which could suggest its participation in the mPTP inhibition observed in IPF fibroblasts.

Diverse studies show that drugs that stabilize the conformation of ANT in the cytosol enhanced mPTP opening, whereas others that stabilize ANT in the matrix inhibited mPTP opening by decreasing the sensitivity to [Ca^2+^]. When the ANT is stabilized in the cytosolic conformation by carboxyattrayloside, it provides a structural basis for mPTP formation by increasing sensibility to [Ca^2+^]. While on the other hand, bongkrekic acid, an inhibitor of ANT that stabilizes the matrix conformation, inhibits mPTP opening by decreasing the sensitivity to [Ca^2+^] [36]. This effect was observed in our results when using attrayloside or bongkrekic acid in the control lung fibroblasts. We observed an increase in apoptosis when stimulated fibroblasts with attrayloside and inhibition with bongkrekic acid.

Other factors that influence the opening of the mPTP may be related to cytochrome c interaction with lipids of the inner mitochondrial membrane or the capacity to accumulate calcium in the mitochondria, as calcium is a well-established mPTP activator. The reduction of mitochondrial calcium release induces mPTP inhibition.

Inhibition of the mPTP augments Ca^2+^ accumulation in mitochondria, stabilizes mitochondrial membrane potential, and defers Ca^2+^ dysregulation [36].

Interestingly, our results on mitochondrial calcium quantification show a marked decrease in mitochondria calcium release in patients with IPF, confirming that mPTP activity decreases in these cells. In addition, we did not observe mitochondrial potential membrane changes, which support that mPTP is inhibited in IPF fibroblasts.

Opening of the mPTP might also stimulate autophagy to eliminate abnormal mitochondria. Thus, the mitochondrial outer membrane recruits the autophagy proteins ATG5 and LC3, not only for mitophagy but also for the anchorage and share of the lipid moieties required for the elongation of the initial phagophore [37]. Interestingly, we have previously found that IPF fibroblasts show a decrease in autophagy caused by activation of the mechanistic target of rapamycin complex 1 (mTORC1) pathway, contributing to the resistance to cell death [38].

As mentioned, we also showed that stimulus with BKA inhibited the mitomycin-induced cell death in control fibroblasts, while the mPTP inductor ATR renders the cell more susceptible to cell death. BKA prevents acidification and is a ligand for the ANT that can inhibit apoptosis. Our results concur with Lui et al., who demonstrated that inhibition of mPTP by cyclosporine A (CsA) and BKA affected p53 translocation in mitochondria, leading to protection against the loss of mitochondrial membrane potential and complex I activity and eventually suppression of apoptosis [39].

We also found that IPF fibroblasts show mitochondrial dysfunction, as evidenced by decreased oxygen consumption and decreased ATP production, although we did not observe significant changes in the ratio ADP/ATP and mitochondrial membrane potential. The fact that the ADP/ATP ratio does not change suggests that the cell adapts to maintain a minimal mitochondrial activity to supply enough ATP and keep the cell alive with basal transmembrane potential, avoiding cell death through the intrinsic pathway of apoptosis.

These findings agree with those previously reported by Álvarez et al. [40], who found low ATP levels in senescent IPF fibroblasts, which were associated with decreased OCR, diminished glycolytic capacity, and abnormalities in oxidative phosphorylation [28,41].

Considerable evidence indicates that the mitochondrial network organization is associated directly with the bioenergetic function and ROS generation. In this sense, our results showed that IPF fibroblasts exhibited a fragmented mitochondrial network and scarce, thinned, and disordered mitochondria with a few electrodense cristae. Diverse studies have shown that cristae architecture is determined by cristae-shaping proteins and depends on the dimeric state of the ATP-synthase [42]. The formation of mitochondrial cristae increases the mitochondrial surface, locating a higher number of enzyme complexes in the mitochondria, improving oxidative phosphorylation (OXPHOS). Alterations in respiration and changes in ATP levels might be associated with mitochondrial morphology modulators. Benard et al. [43] demonstrated that the silencing of DRP1 in HeLa cells resulted in alterations of the mitochondrial network morphology. This effect was associated with reducing sensitivity to apoptosis inducers, potent inhibition of energy production, abnormal connectivity, and multiple budding areas, which suggested a perturbation of mitochondrial dynamics.

Furthermore, we observed a reduction in coupled respiration and ATP production. Studies with the effect of OXPHOS inhibition on mitochondrial network organization in primary human fibroblasts suggest that it may contribute to these abnormalities. Thus, the formation of vesicles due to the inhibition of complex I by rotenone can be regarded as a direct consequence of impaired OXPHOS function [43]. In general, a highly efficient OXPHOS correlates with a highly interconnected and ramified network and enlarged cristae compartments, whereas low OXPHOS activity and high glycolysis correlate with bulkier, more spherical tubules, and isolated mitochondria, displaying reduced intra-cristae space.

We found alterations in the respiratory chain, but we cannot describe it exactly. Therefore, we propose that it is necessary to study the formation of mitochondrial complexes and respirasomes because there is evidence supporting the fact that cytochrome c can influence the function of the chain. Furthermore, morphology observed in IPF fibroblasts increases the evidence that there are issues with the respiratory chain, since the mitochondrial ridges exhibit lesser electro-density than in control fibroblasts. The relationship between mitochondrial morphology and function has gained significant attention in recent years, and it has been verified that there is a direct relationship between the cristae structure and the respiratory chain.

Mitochondria are very complex organelles that control many diverse functions that, as a whole, define the fate of the cell, and mitochondrial dysfunction is usually understood as an event leading to cell death. However, we found that, on the contrary, mitochondrial dysfunction is associated with resistance to apoptosis in IPF fibroblasts, as it occurs in cancer cells. Therefore, the study of the in-depth mechanisms through which mitochondria dysfunction leads to proliferation or death in different pathologies will be helpful to identify new therapeutic targets against IPF, cancer, or other pathologies.

Tissue fibrosis is likely an evolutionarily conserved adaptive process, and persistent fibrosis almost always accompanies incomplete or unsuccessful regeneration. However, in this case, the underlying mechanisms that contribute to the persistence of myofibroblasts and continuous extracellular matrix accumulation in fibrotic tissues remain poorly understood. A growing body of evidence indicates that one of them is the evasion of apoptosis, which usually occurs during physiological wound healing. However, the molecular pathways that are involved in the apoptosis resistance of myofibroblast in fibrotic tissues are still unclear and may even involve different mechanisms. Some evidence indicates the participation of the extrinsic pathway of apoptosis, but no studies had investigated the role of the intrinsic pathway mediated by mitochondria.

In this context, we thought that changes in the mPTP opening, given its relevance in regulating cell death, could be involved. Our results revealed that mPTP is inhibited in IPF fibroblasts, and that calcium, a well-established activator of mPTP, is decreased and pro-apoptotic proteins such as cytochrome c are released as well. We consider that this finding is relevant to understand some of the biopathological mechanisms that participate in the apoptosis resistance of fibroblasts during progressive fibrosis as in IPF. A better understanding of the molecular composition of the mPTP and apoptosis mechanisms will provide clues for effective and selective therapeutic strategies for the treatment of this devastating disease.

## 4. Materials and Methods

### 4.1. Reagents

We purchased the Cell Proliferation Reagent WST-1 (11644807001) from Roche Diagnostics, Mannheim, Germany. The FITC Annexin V Apoptosis Detection Kit (556547) was from BD Biosciences, San Diego, CA, USA. The Mitochondria Isolation Kit for Cultured Cells (89874), Pierce™ BCA Protein Assay Kit (23225), HRP-anti-goat IgG (611620), and Anti-GAPDH (PA1-987) were from Thermo Fisher Scientific, Rockford, IL, USA. The Image-IT™ LIVE Mitochondrial Transition Pore Assay Kit (I35103), MitoProbe™ Transition Pore Assay Kit (M34153), ATP Determination Kit (A22066), and tetraethyl benzimidazolyl carbocyanine iodide dye JC-1 (T3168) were from Molecular Probes, Eugene, OR, USA. The ADP/ATP ratio assay kit (MAK135), anti-β-actin (A5441), oligomycin A, rotenone, antimycin A, L-glutamic acid, L( )-malic acid disodium salt, sodium succinate dibasic hexahydrate, carbonyl cyanide 4-(trifluoromethoxy) phenylhydrazine (FCCP), carbonyl cyanide-3-chlorophenyl hydrazone (CCCP), ethylene glycol-bis (2-aminoethylether)-N,N,N′,N′-tetra acetic acid (EGTA), potassium salt, 4-(2-hydroxyethyl)piperazine-1-ethanesulfonic acid potassium salt (HEPES), bovine serum albumin (BSA), free fatty acid, magnesium chloride (MgCl2), potassium phosphate monobasic (KH2PO4), cytochrome c, bongkrekic acid (BKA), and atractyloside (ATR) were purchased from Sigma-Aldrich from St. Louis, MO, USA. Mitomycin C (sc-3514); anti-VDAC-1 (sc-390996), and anti-caspase-3 (sc-7272) were from Santa Cruz Biotechnology, Heidelberg, Germany. Anti-cytochrome c (612504) and HRP-anti-rabbit IgG (406401) were from BioLegend, San Diego, CA, USA. The anti-caspase-9 (ab32539) was from Abcam Cambridge, MA, USA. HRP-Anti-Mouse IgG (115-035-003) was acquired from Jackson Immuno Research, West Grove, PA, USA.

### 4.2. Bioethics and Experimental Design

The Bioethics Committee of the Instituto Nacional de Enfermedades Respiratorias approved this research, with ethic approval code B05-21, (20 September 2018). Fibroblasts from IPF patients were obtained from lung biopsies performed for diagnostic purposes; all participants provided informed consent, and their personal and identification data were protected. As controls, healthy lung fibroblasts were obtained by lobectomy from patients without morphological data of interstitial disease, and the commercial human normal fibroblasts line NHLF (Normal Human Lung Fibroblasts CC-2512 Lonza Clonetics) was used. Healthy and IPF lung fibroblasts matched by age were studied under basal conditions and after mitomycin C-induced apoptosis.

### 4.3. Cell Culture

The fibroblasts were cultured in Ham medium (F-12), supplemented with penicillin (100 U/mL), streptomycin (100 mg/mL), and 10% fetal bovine serum (SFB), and incubated in a humid atmosphere at 37 °C with 5% CO_2_, until 80% confluence. Fibroblasts were treated with different doses of mitomycin C (10, 25, or 50 μg/mL) in F-12 medium with 1% SFB at different times (4, 8, 16, and 24 h). Control fibroblasts were incubated in F-12 medium with 1% SFB for the same period. After the treatment, fibroblasts were washed three times with phosphate-buffered saline (PBS) and kept in the F-12 medium for subsequent experiments.

### 4.4. Cell Viability

Cell viability was assessed using the Cell Proliferation Reagent WST-1 assay following the manufacturer’s instructions. Cells were seeded in 96-well plates at a final density of 5 × 10^3^ cells per well in 100 μL of culture medium. After treatment with mitomycin-C, 10 μL of WST-1 solution was added to each well, and the cells were incubated for 3 h at 37 °C in a 5% CO_2_ atmosphere. The optical density was measured using a multimodal microplate reader (Synergy HTX BioTek, Winooski, VT, USA) at a wavelength of 450 nm.

### 4.5. Cell Apoptosis

Fibroblasts were stimulated with mitomycin-C for 24 h and stained with Annexin V-IP for flow cytometric analysis. Staurosporine (1 μM) was used as a positive control. Cells were acquired using a FACSAria flow cytometer (BD Biosciences). Data were analyzed using FlowJo 7.8 software (FlowJo, Ashland, OR, USA, Becton Dickinson and Company).

### 4.6. Preparation of the Cytosolic and Mitochondrial Fractions

Control and IPF fibroblasts were cultured in a cell culture flask of 175 cm^2^ until 80% confluence (approximately two or three flasks by experimental condition). Fibroblasts were stimulated with mitomycin C (25 μg/mL) for 4 and 24 h. After treatment with mitomycin C, the cells were washed and trypsinized, and their viability was analyzed by a Trypan Blue exclusion test. For each experimental condition, 1 × 10^7^ viable cells were used.

The isolation of mitochondrial and cytosolic fractions of fibroblast cultures was performed with the Mitochondria Isolation Kit for Cultured Cells, following the manufacturer’s instructions. For this assay, we used a Dounce homogenizer to disrupt cells, and subsequently, we separated the fractions by differential centrifugation. The mitochondrial and cytosolic fractions were used for analysis by Western blotting. The mitochondrial pellet was lysed with 50 μL of 2% detergent 3-(3-cholamidopropyl) dimethylammonio)-1-propane sulfonate (CHAPS) in Tris buffer saline (TBS; 25 mM Tris, 0.15 M NaCl; pH: 7.2). The supernatant contained the soluble mitochondrial protein that was quantified by the Pierce BCA Protein Assay Kit.

### 4.7. Western Blotting

Cytosolic and mitochondrial fractions and cell lysates were analyzed by Western blot. Cellular lysates from human normal and IPF fibroblasts were prepared using radioimmunoprecipitation assay buffer (RIPA) with phenylmethylsulfonyl fluoride (PMSF) and protease inhibitors. The protein concentration was quantified using the Bradford assay. Briefly, 16 μg of protein were separated by Sodium Dodecyl Sulfate-Polyacrylamide SDS-PAGE (110 V for 1 h at room temperature) and transferred onto a nitrocellulose membrane using a wet chamber blotting system. The nitrocellulose membranes were blocked with 5% skim milk for 1 h and afterwards incubated overnight at 4 °C with the primary antibodies: anti-cytochrome c (1:500); anti-caspase 9 (1:500); anti-VDAC-1 (1:100); anti-Bax (1:100); anti-ATP5A (1:500); anti-ANT1 (1:1000); anti-GAPDH (1:2000); anti-β-actin (1:10,000). The secondary antibodies were incubated for 1 h at room temperature with the following dilutions: anti-mouse (1:10,000), anti-rabbit (1:2500), and anti-goat (1:1000).

### 4.8. Cytochrome C Release

The cytosolic fraction was filtered through a polyvinylidene fluoride membrane PVDF (0.45 μm), and the cytochrome c content was determined by high-performance liquid chromatography (HPLC) using a 300 Å Delta Pack C4 column with 5 μm particles (3.9 × 150 mm; Waters). A gradient of 30% acetonitrile in water with trifluoroacetic acid (0.1% *vol*/*vol*) to 70% acetonitrile in water with trifluoroacetic acid (0.1% *vol*/*vol*) over 15 min with a flow rate of 1 mL/min was used as was described elsewhere [44]. Cytochrome c was detected at 393 nm, and its concentration was quantified using a standard curve of cytochrome c from bovine heart.

### 4.9. mPTP Opening

The mPTP opening was assessed by cobalt quenching of calcein-AM fluorescence. After treatment, cells were loaded with calcein-AM (1 μM, Molecular Probes, Life Technologies) at 37 °C in the dark, CoCl_2_ (1 mM) was added, and cells were incubated for another 15 min. Then, the fluorescence of 30,000 cells for each experiment was measured with a flow cytometer (FACS Aria II, BD, San Jose, CA, USA), and the data were processed with the FlowJo software. For imaging experiments, 1 × 10^4^ fibroblasts were cultured on glass-bottom culture dishes. First, cells were loaded with calcein-AM (1 μM) and MitoTracker Red (150 nM), with or without CoCl_2_ (1 mM), in Hanks’ Balanced Salt Solution (HBSS) 1X for 15 min at 37 °C. Then, cells were washed three times with HBSS 1X. Live images of the cells were captured with the Olympus FluoView FV1000 Confocal Microscope and analyzed using the ImageJ software version 1.53i (Bethesda, MD, USA).

### 4.10. Intracellular Calcium Concentration Assay ([Ca^2+^]_i_)

To evaluate [Ca^2+^]_i_ changes induced by trifluoromethoxy carbonyl cyanide phenylhydrazone (FCCP) in control and IPF fibroblasts, cells were plated in round coverslips coated with poly-L-Lysine and cultured for 2 days. Then, cells were loaded with Fura 2-AM (2.5 μM) in a low concentration of Ca^2+^ (0.1 mM) and at room temperature. Then, fibroblasts were incubated for 1 h at 37 °C under a 5% CO_2_ atmosphere. Afterward, cells were transferred to a heated perfusion chamber (37 °C) mounted on an inverted Nikon Diaphot 200 microscope (Nikon, Tokyo, Japan). Cells were recorded under continuous perfusion and carbogen bubbling (to maintain pH at 7–4) at a rate of 2–2.5 mL/min with Krebs–Ringer buffer (NaCl 118 mM, NaHCO_3_ 25 mM, KCl 4.7 mM, KH_2_PO_4_ 1.2 mM, MgSO_3_ 1.2 mM, glucose 11 mM, and CaCl_2_ 2.5 mM) at 37 °C. After the recording of basal fluorescence, cells were exposed to FCCP 1 μM. Next, fibroblasts loaded with Fura 2-AM were alternately submitted to Xe lamp at 340 nm and 380 nm excitation light, and the emission fluorescence was measured at 510 nm using a microphotometer (model D-104), from Photon Technology International (PTI, Princeton, NJ, USA). Fluorescence was measured at intervals of 0.5 s for 10 min, and the intracellular Ca^2+^ concentration ([Ca^2+^]_i_) was calculated according to the Grynkiewicz [45] formula as follows:

[Ca^2+^]_i_ = bKd (R − R_min_)/(R_max_ − R)

where Kd is the dissociation constant of Fura-2AM, b is the ratio of fluorescent signals (R) at 380 nm for Ca^2+^-free and Ca^2+^-saturated dye, R_min_ is R in the absence of external Ca^2+^, and R_max_ is R in saturating [Ca^2+^]_i_ [46]. These parameters were determined in vitro. The mean 340–380 nm fluorescence ratios for R_max_ (6.06) and R_min_ (0.39) were obtained by exposing the cells to Ca^2+^ (10 mM) in the presence of ionomycin (10 μM), and Ca^2+^ free Krebs with EGTA (10 mM), respectively. The fluorescence ratio at 380 nm light excitation in Ca^2+^ free medium and Ca^2+^ saturated cells was 4.23. The Kd of Fura 2-AM was assumed to be 386 nM [46]. To evaluate the Ca^2+^ response to FCCP, cells were perfused with 1 μM FCCP to stimulate the mitochondrial permeability transition pore (mPTP). To pharmacologically determine if mPTP were involved in this response, a blocker cyclosporin A (CspA) 10 μM plus ATP was used.

### 4.11. Cell Respirometry

#### 4.11.1. Respirometry in Non-Permeabilized Cells

The oxygen consumption experiments in intact cells were performed as previously described using a high-resolution respirometry O2 k meter (Oroboros Instruments, Innsbruck, Austria) [47]. Briefly, measures were made at 37 °C using 2 mL of culture medium. The respiratory parameters were defined as (a) basal respiration, corresponding to the oxygen consumption in the presence only of cells, (b) the leak of the respiration, corresponding to cellular oxygen consumption in the presence of 5 μM oligomycin, (c) respiratory control corresponding to the ratio basal/leak, (d) the maximum uncoupled respiration was achieved by titrations of 0.5 μL of 1 μM CCCP. All parameters were normalized by the number of cells and corrected by the non-mitochondrial residual respiration, which was obtained by adding 5 μM rotenone plus 5 μM antimycin A.

#### 4.11.2. Respirometry in Permeabilized Cells with Digitonin

The oxygen consumption in permeabilized fibroblasts was performed as was previously described by Kuznetsov et al. [48]. Control and IPF fibroblasts were seeded in standard conditions, and the trypan blue exclusion test was used to determine cell density. For each experimental condition, 5 × 10^5^ fibroblasts were added, and the system was equilibrated again for 5 min. The reaction was carried out in respiration medium (EGTA 0.5 mM; MgCl_2_ 3 mM; KH_2_PO_4_ 10 mM; HEPES 20 mM; BSA 1 g; mannitol 110 mM for liter) pH 7.1, adjusted with 5 N KOH. Next, the fibroblasts were permeabilized with digitonin (20 μg/mL) and incubated for 5 min at 37 °C. After the addition of digitonin, the respiration rate should markedly decline for 3–5 min. Then, glutamate/malate (10 mM/5 mM) was used as a substrate for complex I activity, ADP was added to induce ATP production, and the respiration was inhibited by 0.5 μM rotenone, which is a specific inhibitor of complex I. Afterward, succinate (10 mM) was used as a substrate to induce complex II supported respiration, ADP was added to induce ATP production, and respiration was inhibited with oligomycin (5 μM) (state 4). Finally, 2 mM ADP was added for maximal (state 3) mitochondrial respiration. The respiratory control ratio (RCR) was calculated by dividing state 3/state 4 respiratory values.

### 4.12. Mitochondrial Membrane Potential (Δψm)

Mitochondrial Δψ was measured using JC-1, which is a fluorescent dye that exhibits potential-dependent accumulation in mitochondria. Polarized mitochondria are marked by punctate orange-red fluorescent staining. On depolarization, the orange-red punctate staining is replaced by diffuse green monomer fluorescence. Thus, mitochondrial depolarization is indicated by a decrease in the red/green fluorescence intensity ratio. Briefly, IPF and control fibroblasts were stained with JC-1 in Ham F-12 culture medium for 30 min at 37 °C in the dark. Then, cells were washed twice with PBS and stimulated with the uncoupling agent FCCP (100 μM) or oligomycin (5 μM). Fibroblasts were observed with a confocal microscope using the 20x objective, and both green (520 nm) and red (572 nm) fluorescence were measured to detect the emission shift. This shift was calculated as the red/green fluorescence intensity ratio. Images were analyzed using ImageJ software.

### 4.13. ATP Production

ATP production was determined quantitatively in IPF and control fibroblasts using the ATP Determination Kit, which uses a bioluminescent assay. For this assay, 10,000/well cells were plated on a white 96-well microplate with a clear bottom, and the assay was performed following the manufacturer’s instructions. The bioluminescence was recorded using a multimodal microplate reader (Synergy HTX BioTek, Winooski, VT, USA) in the luminometer mode.

### 4.14. ADP/ATP Ratio

The ADP/ATP ratio in IPF and control fibroblasts was measured using the ADP/ATP ratio assay kit. Briefly, IPF and control fibroblasts were plated at a density of 10,000 cells/cm^2^ in a white 96-well microplate with a clear bottom. Following overnight incubation, the ADP/ATP ratio was determined following the manufacturer’s guidelines. Next, the bioluminescence was measured using a multimodal microplate reader (Synergy HTX BioTek, Winooski, VT, USA).

### 4.15. Transmission Electron Microscopy (TEM)

TEM was done with the support of the Center for Advanced Microscopy (Northwestern University). In brief, lung fibroblasts were cultured on Thermanox coverslips placed in a 24-well plate. After 48 h of incubation, samples were fixed in 0.1 M sodium cacodylate (pH 7.2) containing 2% paraformaldehyde and 2.5% glutaraldehyde and post-fixed with 2% osmium tetroxide and stained with 3% uranyl acetate. Samples were dehydrated in ascending ethanol grades, transitioned with a 1:1 mixture of ethanol and resin, and embedded in the resin mixture of the EMbed-812 kit. Using a Leica Ultracut UC6 ultramicrotome, ultra-thin sections (70 nm) were collected on 200 mesh copper grids and post-stained with 3% uranyl acetate and Reynolds lead citrate. Ultra-thin sections were used to obtain images from each sample with the Tecnai G2 Spirit transmission electron microscope (FEI Company, Hillsboro, OR, USA) at 120 kV. The length of mitochondria was used to determine mitochondrial elongation mean per image; at least 70 mitochondria per condition were measured. Images were analyzed using the ImageJ software.

### 4.16. Statistical Analysis

All the data were expressed as the mean ± standard error mean (SEM). Two-way or one-way analysis of variance (ANOVA) followed by Bonferroni test assessed the significance of the differences. To analyze the significant differences between the control and IPF group, we used an unpaired *t*-test. *p*-values of less than 0.05 were considered to be statistically significant. Statistical analysis was performed using the GraphPad Prism 5.01 software (San Diego, CA, USA).

## Data Availability

All the data are included in the manuscript.
